# Network Pharmacology-Based Analysis of the Pharmacological Mechanisms of Aloperine on Cardiovascular Disease

**DOI:** 10.1155/2020/5180716

**Published:** 2020-07-14

**Authors:** Bingwu Huang, Juncheng Xiong, Xuyong Zhao, Yihan Zheng, Ning Zhu

**Affiliations:** ^1^Department of Anesthesiology, The Wenzhou Third Clinical Institute Affiliated to Wenzhou Medical University, Wenzhou People's Hospital, No. 299 Guan Road, Wenzhou 325000, Zhejiang Province, China; ^2^Department of Cardiology, The Wenzhou Third Clinical Institute Affiliated to Wenzhou Medical University, Wenzhou People's Hospital, No. 299 Guan Road, Wenzhou 325000, Zhejiang Province, China; ^3^Eye Hospital, School of Ophthalmology & Optometry, Wenzhou Medical University, State Key Laboratory of Ophthalmology, Optometry and Visual Science, No. 270 Xueyuan West Road, Wenzhou 325000, Zhejiang Province, China

## Abstract

**Background:**

Aloperine is an active component of *Sophora alopecuroides* Linn, which has been extensively applied for the treatment of cardiovascular disease (CVD). However, our current understanding of the molecular mechanisms supporting the effects of aloperine on CVD remains unclear.

**Methods:**

Systematic network pharmacology was conducted to provide testable hypotheses about pharmacological mechanisms of the protective effects of aloperine against CVD. Detailed structure was obtained from Traditional Chinese Medicines Integrated Database (TCMID). Target genes of aloperine against CVD were collected from SwissTargetPrediction, DrugBank database, and Online Mendelian Inheritance in Man (OMIM) database. Gene Ontology (GO) enrichment analysis, Kyoto Encyclopedia of Genes and Genomes (KEGG) pathway performance, and network construction were adopted to explore common target genes.

**Results:**

Our findings showed that 25 candidate targets were the interacting genes between aloperine and CVD. GO analysis revealed biological process, cellular component, and molecular function of these target genes. More importantly, the majority of enrichment pathways was found to be highly associated with the nitrogen metabolism by KEGG analysis. Core genes particularly in nitrogen metabolism pathway including carbonic anhydrase (CA) III, CA IV, CA VA, CA VB, CA VI, CA VII, CA IX, CA XII, and CA XIV can be modulated by aloperine in the nitrogen metabolism.

**Conclusion:**

Our work revealed the pharmacological and molecular mechanisms of aloperine against CVD and provided a feasible tool to identify the pharmacological mechanisms of single active ingredient of traditional Chinese medicines.

## 1. Introduction

Though great progress toward the eradication of cardiovascular disease (CVD) has been achieved, the related issues of disease prevention and cure have been elusive [1]. CVD remains the leading cause of death all over the world. More than 95% of cardiovascular deaths can be attributed to ischemic heart disease, stroke, heart failure, cardiomyopathy, rheumatic heart disease, and atrial fibrillation [2]. Although Western medicines which can reduce cardiovascular morbidity and mortality in at-risk individuals or those with established disease are used in clinical setting, the risks of adverse effects from these medicines are also considerable [3]. Therefore, strategies that develop new drugs are urgently needed for CVD therapies. Traditional Chinese medicine (TCM) has evolved over thousands of years, with both unique theories and rich experience. TCM medications has been used as a complementary and alternative approach for primary and secondary prevention of CVD [4]. However, further studies should be conducted to evaluate the mechanisms of preventive effects of TCM on CVD.

Aloperine is extracted from the traditional Chinese medicine *Sophora alopecuroides* L., which possesses a variety of pharmacological activities such as anti-inflammatory, antifibrosis, anticancer, and antimicrobial [5]. Aloperine executes antitumor effects through the induction of apoptosis and cell cycle arrest in vitro and in vivo [6, 7]. Considerably, the biological properties and potential therapeutic mechanisms of aloperine have been widely tested in laboratory-based studies [8–11]. However, a systematical understanding of how the multiple therapeutic targets work together to exert therapeutic effects on CVD still needs further investigation.

**Figure 1:**
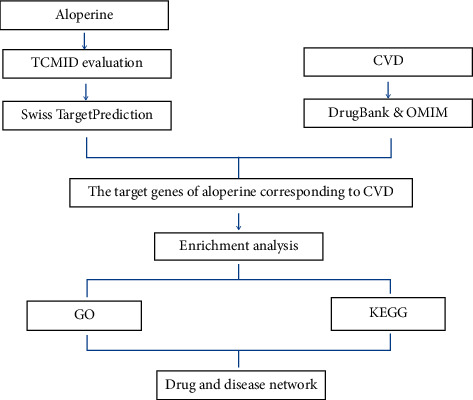
A schematic diagram based on systematic pharmacological strategy for revealing molecular mechanisms of aloperine acting on CVD.

Network pharmacology is a comprehensive method to uncover the complex network relationships between TCM and diseases [12]. This approach can integrate the various databases of TCM, proteins, genes, and pathways for analysis and construct a drug-target-disease network. In addition, the network pharmacology approaches have been successfully used to analyze the main active components and identify the potential therapeutic mechanisms of TCM involved in their curative effects [13]. In the present study, we systematically elucidated the mechanisms of therapeutic effects of aloperine on CVD by the network pharmacology approach. The drug structure of aloperine was evaluated by using TCMID. Then, the potential candidate targets of aloperine and the CVD-related targets were predicted by SwissTargetPrediction [14] and collected from DrugBank database and Online Mendelian Inheritance in Man (OMIM) database [15, 16], respectively. Furthermore, the identified targets were screened for the analysis of target genes enrichment and pathway. Finally, the drug-target-pathway network was constructed to provide a systematic overview of the potential pharmacological mechanisms of aloperine on CVD. The schematic diagram is shown in Figure 1.

## 2. Materials and Methods

The structure of aloperine was explored by using TCMID database (http://www.megabionet.org/tcmid/), which is a comprehensive database aiming at TCM's modernization and standardization. It has been highly recognized among pharmacologists and scholars in TCM researches [17]. TCMID stores records about >8000 herbs, 25000 herbal compounds, 17500 targets, and other related information for TCM as well as modern medicine, which is the largest data set for related field [18]. In the present study, the chemical name “aloperine” was input into the ingredient search box and its molecular structural formula was revealed.

### 2.1. Target Identification of Aloperine

The aloperine targets were collected from the SwissTargetPrediction (http://www.swisstargetprediction.ch), which is a network tool designed to accurately predict targets for bioactive molecules [14]. In the SwissTargetPrediction current version with default parameters, the potential candidate target prediction was performed by inputting aloperine SMILES and searching for their similar molecules [19]. The screening condition was limited to “*Homo sapiens*” and high probability targets (probability *P* < 0.05) were collected after duplicate contents were eliminated.

### 2.2. Target Identification of Known Therapeutic Targets Acting on CVD

The CVD-related therapeutic targets were found from the following two databases. One is the DrugBank (http://www.drugbank.ca), which is a comprehensive, freely available web resource with molecular information about drugs, drug targets, drugs action, and drugs interaction [15]. “Cardiovascular diseases” were searched in DrugBank, and those targets, which are FDA- (Food and Drug Administration-) approved CVD-related drug targets and human genes/proteins, were selected. Another is the OMIM database (https://omim.org), which is a comprehensive database of genes, genetic phenotypes, and their relationships [16]. The query “Cardiovascular disease” was used as the keyword to search for CVD-related targets in the OMIM database.

### 2.3. Analysis by GeneMANIA

GeneMANIA (http://genemania.org), a flexible and user-friendly website, is mainly used to generate hypotheses about gene functions, analyze gene lists, and determine gene priority for function determination [20]. GeneMANIA can show genes with similar functions through a large number of genomic and proteomic data. The potential candidate target genes were input into the search box after selecting *Homo sapiens* from the organism option. And a functional relationship network revealed protein domains and genetic interactions.

### 2.4. Gene Function and Pathway Enrichment Analysis

WebGestalt (http://www.webgestalt.org) can be used to further understand the function and pathway enrichment information of related genes. In the current version, WebGestalt is rich in 12 kinds of organisms, 342 kinds of gene identifiers, and 155175 kinds of functional classifications, as well as functional databases uploaded by other users. The candidate target genes were input into the Gene Ontology (GO) database and Kyoto Encyclopedia of Genes and Genomes (KEGG) databases of WebGestalt server, respectively, and the Over Representation Analysis (ORA) method was selected for analysis.

Go analysis can classify individual genomic products (e.g., genes, proteins, ncRNAs, and complexes) and create evidence-supported annotations to describe the biological functions, including molecular function, biological pathways, and cellular components [21]. KEGG is a database of genome deciphering, which can give functional meaning to genes and genomes at molecular and higher levels [22]. False discovery rate- (FDR-) adjusted *P* value was calculated in these two enrichment analyses, and *P* < 0.05 indicates the enrichment degree had statistical significance and the pathway results would certainly be necessary functional mechanisms of CVD.

### 2.5. Network Construction

Cytoscape offers a versatile and interactive visualization interface for exploring biomedical networks composed of proteins, genes, and other types of interactions, which can promote research tasks such as predicting gene functions and building pathways [23]. In this study, Cytoscape software (version 3.6.1, Boston, MA, USA) was utilized to establish three-layer networks for better understanding of the complex relationship among compounds, target genes, and diseases.

## 3. Results

### 3.1. Structure of Aloperine

**Figure 2:**
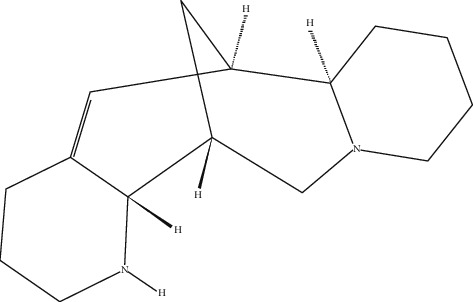
The chemical structure of aloperine downloaded from the PubChem database (CID: 162147).

The detailed structure of aloperine was provided by TCMID. The molecular formula of aloperine is C_15_H_24_N_2_ and SMILES is C1CCN2CC3CC(C2C1)C=C4C3NCCC4. The structural diagram of the chemical name “aloperine” are shown in Figure 2.

### 3.2. Targets Identification of Aloperine and CVD

The aloperine SMILES was put into the SwissTargetPrediction and the predicted targets were collected. A total of 102 candidate target genes were identified by Swiss Target Prediction (Supplementary Table (available here)). 76 known CVD target genes were obtained from the DrugBank database and 474 known CVD-related target genes were found from the OMIM database. Then, 538 known CVD target genes are identified by eliminating the repeated CVD target genes (Supplementary Table). Finally, we compared the target genes of CVD and aloperine, and the 25 same target genes were collected (Table 1). These 25 identified interacting genes were chosen for further investigation.

### 3.3. GeneMANIA Analysis

**Figure 3:**
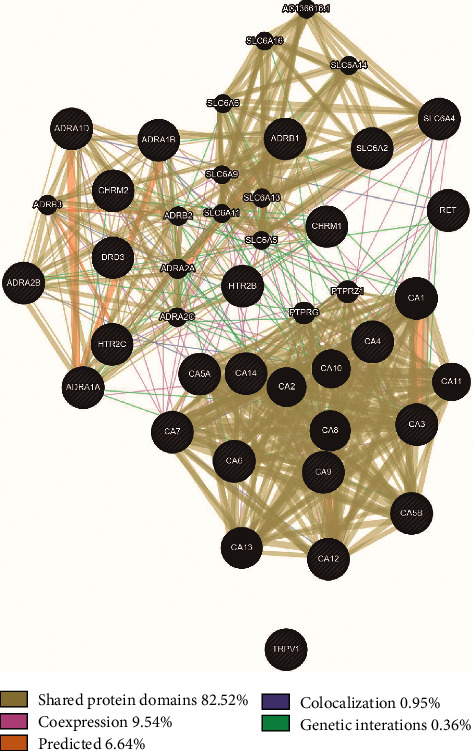
Protein network of aloperine. Functional associations between targets were investigated using Gene MANIA. Black nodes indicated target proteins and connecting colors suggested different correlations. Genes in black circles were query terms while these in gray circle represented genes associated with query genes.

Among the 25 target genes and their interacting proteins, it was found that 82.52% had shared protein domains, 9.54% had coexpression, 6.64% had predicted, 0.95% had colocalization, and 0.36% had genetic interactions (Figure 3).

### 3.4. GO and Pathway Analysis

**Figure 4:**
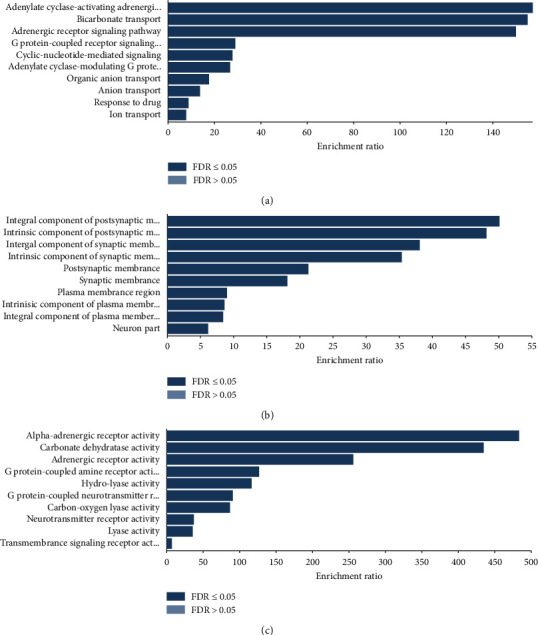
GO map of putative target genes. (a) Biological process categories. (b) Cellular component categories. (c) Molecular function categories.

The 25 identified target genes were analyzed by using WebGestalt for GO and KEGG analysis. An introduction of the GO analysis was discovered with the enriched conditions in the biological process, cellular component, and molecular function categories (Figure 4). Depending on the outcomes of GO enrichment, the enriched biological process categories were dominated by adenylate cyclase-activating adrenergic receptor signaling pathway, bicarbonate transport, and adrenergic receptor signaling pathway. Cell component analysis showed that integral component of plasma membrane and intrinsic component of plasma membrane mainly accounted for the largest proportion. The enriched molecular function categories were dominated by alpha-adrenergic receptor activity and carbonate dehydratase activity.

**Figure 5:**
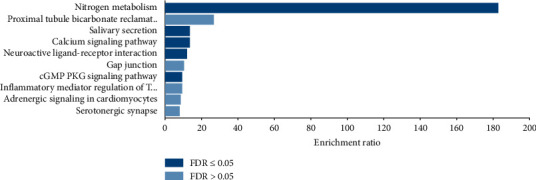
KEGG pathway analysis of putative target genes.

The pathway analysis showed the 25 target genes were enriched in nitrogen metabolism, salivary secretion, calcium signaling pathway, neuroactive ligand-receptor interaction, and cGMP-PKG signaling pathway (Figure 5).

### 3.5. Network Analysis

**Figure 6:**
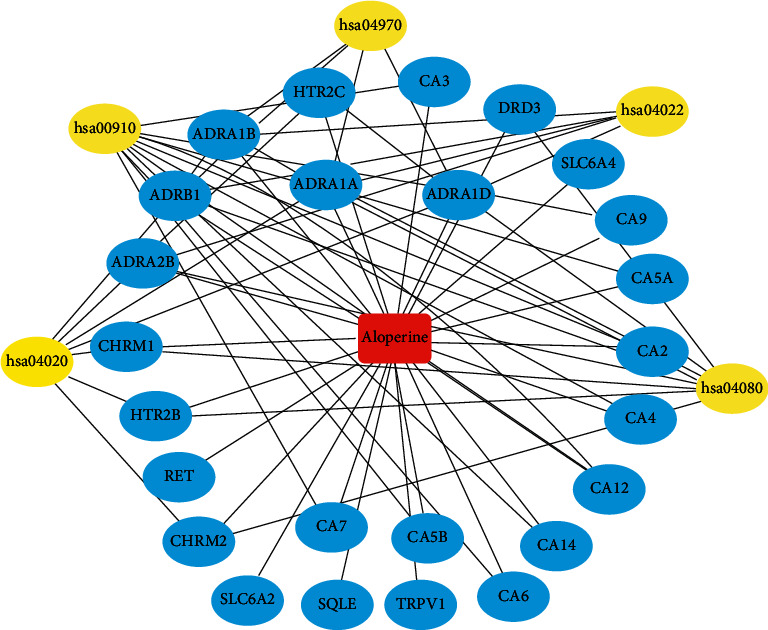
Aloperine-target-pathway network.

An entire network of compound, target genes, and pathway was constructed by using Cytoscape (v 3.6.1) according to target and pathway analyses. This network has 32 nodes and 63 edges (Figure 6). The red oblong, blue circles, and yellow circles represented aloperine, target genes, and pathways, respectively.

## 4. Discussion

TCM has developed over thousands of years to treat and prevent many human diseases; however the underlying mechanisms of TCM at the molecular level and a systems perspective remain great challenges [13]. The network pharmacology approaches focus on exploring the network connectivity, drug targets, and designing the optimal therapeutic strategies, the emergence of which can uncover the underlying complex relationships between TCM and diseases by the prediction of gene targets [24]. In silico method used in the present study contributed to the better understanding of the molecular mechanisms of the pharmacological effects of aloperine on CVD and the aloperine-based drug discovery.

In the present report, 25 candidate target genes were determined as interacting genes between aloperine and CVD. Our GeneMANIA analysis revealed 82.52% had shared protein domains. The data also showed 9.54% had coexpression, 6.64% had predicted, 0.95% had colocalization, and 0.36% had genetic interactions. These target genes mainly having shared protein domains indicate that these genes share similar biological functions.

To evaluate the biological processes, cellular components, and molecular functions of aloperine, we analyzed the candidate targets by performing GO enrichment analysis. GO analysis revealed that target genes were majorly associated with the biological process of bicarbonate transport and molecular function of carbonate dehydratase activity. To further identify that the biological process and molecular function were involved in the corresponding pathological events during the progression of CVD, KEGG pathway enrichment analysis was conducted. KEGG analysis suggests that aloperine may exert therapeutic effects against CVD by regulating the nitrogen metabolism, salivary secretion, calcium signaling pathway, neuroactive ligation-receptor interaction, and cGMP-PKG signaling pathway. In line with GeneMANIA and GO analyses, the nitrogen metabolism was highly enriched in KEGG pathway analysis, which indicated that aloperine may exert therapeutic effects on CVD mainly through the nitrogen metabolism. In addition, the nitrogen metabolism can also be considered as an efficient pathway for CVD treatment. In fact, it was well known that nitrogen metabolism plays a crucial role in CVD therapeutics, especially the NO-sGC-cGMP pathway and the use of nitrates [25].

Our data showed other gene targets, including carbonic anhydrase (CA) III, CA IV, CA VA, CA VB, CA VI, CA VII, CA IX, CA XII, and CA XIV involved in the nitrogen metabolism were found to exert therapeutic effects on CVD. GO enrichment analysis of cell component showed integral component of plasma membrane and intrinsic component of plasma membrane mainly accounted for the largest proportion. As is known, CA IV, CA VI, CA IX, CA XII, and CA XIV are localized in plasma membrane [26]. Furthermore, consistent with GeneMANIA analysis, these CAs have a catalytic domain. CAs participate in a variety of biological processes, including the exchange of carbon dioxide in tissues and alveoli and the formation of a number of body fluids [27]. CAs are a pharmacological target and inhibitors of this enzyme used to treat a number of diseases including CVD [28]. CA inhibitor such as ethoxzolamide was previously used as diuretics for the treatment of hypertension and heart failure [29]. CA IV and CA XIV are localized in sarcoplasmic reticulum membrane and the sarcolemmal membrane, and CA IX is located in the terminal sarcoplasmic reticulum-tubular region [30]. Transmembrane CA IX forms a complex with Na^+^/HCO_3_^−^ cotransporter (NBC) e1, leading to activating NBCe1-mediated HCO_3_^−^ influx in cardiac muscle [31]. Hypoxia causes the expression of membrane-associated CA IX isoform increased by 60% in hearts with cardiac dysfunction in rat, which is blocked by its inhibitor—diffusible ethoxzolamide [32]. The increase of the expression CAIV is a marker of the hypertrophic human heart and contributes to heart failure [33]. A CA IX inhibitor (benzolamide) reduces myocardial infarction and prevents myocontractile function [34]. In addition, CA IX also plays a critical role in vessel functions. It is also reported that acetazolamide, a CA IX inhibitor, attenuates hypoxic pulmonary vasoconstriction, improves the subendocardial oxygen supply-demand ratio, and increases hemoglobin-oxygen saturation [35, 36]. CA IX modulates pulmonary microvascular endothelial cell pH and angiogenesis during acute lung injury and acidosis [37]. The expression of CA XIV is upregulated in cardiomyocytes of spontaneously hypertensive rats compared to normal hearts [38]. CA IX is a critical determinant of CVD and a promising drug target, which is also targeted by aloperine. Though the effects of these CAs have been investigated in CVD treatment, further research of CAs acting on CVD and development of the novel modulation of nitrogen metabolism pathway on CVD needs to be promoted.

The challenges of applying network pharmacology lie primarily with the lack of interpretability and repeatability of these results. Systematic and comprehensive experimental data still need to be performed. In the present study, the mechanisms of protective effects of aloperine on CVD were explored. However, the pathogenesis of CVD is complex and CVD includes a variety of different disease. Hence, further experiments should be performed to verify the conclusions and these targets also needed to be validated in different disease. With ongoing efforts to tackle these issues, the application of network pharmacology still can promote data-driven decision-making, speed up the process, and reduce the costs in drug discovery and development.

## 5. Conclusion

In the present study, we performed a network pharmacology coupled to unveil the chemical basis and investigate the action mechanisms of aloperine for treating CVD. Our data showed 25 identified interacting genes were involved in the protective effects of aloperine on CVD. In addition, our results predict these therapeutic targets are mainly associated with regulation of the nitrogen metabolism and nine key targets were identified, including CA III CA IV, CA VA, CA VB, CA VI, CA VII, CA IX, CA XII, and CA XIV. Our research may contribute further work to identify the mechanism of aloperine on CVD and the central role of CAs on CVD, which result in new drug discovery.

## Figures and Tables

**Table 1:** Target genes of aloperine related with CVD.

Number	Gene ID	Gene symbol	Gene name
1	6530	SLC6A2	Solute carrier family 6 member 2
2	3358	HTR2C	5-Hydroxytryptamine receptor 2C
3	3357	HTR2B	5-Hydroxytryptamine receptor 2B
4	151	ADRA2B	Adrenoceptor alpha 2B
5	146	ADRA1D	Adrenoceptor alpha 1D
6	148	ADRA1A	Adrenoceptor alpha 1A
7	147	ADRA1B	Adrenoceptor alpha 1B
8	5979	RET	Ret proto-oncogene
9	153	ADRB1	Adrenoceptor beta 1
10	766	CA7	Carbonic anhydrase 7
11	761	CA3	Carbonic anhydrase 3
12	765	CA6	Carbonic anhydrase 6
13	771	CA12	Carbonic anhydrase 12
14	23632	CA14	Carbonic anhydrase 14
15	768	CA9	Carbonic anhydrase 9
16	762	CA4	Carbonic anhydrase 4
17	11238	CA5B	Carbonic anhydrase 5B
18	763	CA5A	Carbonic anhydrase 5A
19	1129	CHRM2	Cholinergic receptor muscarinic 2
20	1128	CHRM1	Cholinergic receptor muscarinic 1
21	1814	DRD3	Dopamine receptor D3
22	6532	SLC6A4	Solute carrier family 6 member 4
23	7442	TRPV1	Transient receptor potential cation channel subfamily V member 1
24	760	CA2	Carbonic anhydrase 2
25	6713	SQLE	Squalene epoxidase

## Data Availability

The data used to support the findings of this study are included within the article.

## Supplementary Materials

Supplementary Materials Known targets of aloperine and known therapeutic targets acting on CVD.
